# Iron-free and iron-saturated bovine lactoferrin inhibit survivin expression and differentially modulate apoptosis in breast cancer

**DOI:** 10.1186/s12885-015-1441-4

**Published:** 2015-05-22

**Authors:** Jessica A. Gibbons, Jagat R. Kanwar, Rupinder K. Kanwar

**Affiliations:** Nanomedicine - Laboratory for Immunology and Molecular Biomedical Research, Molecular and Medical Research Facility, School of Medicine, Faculty of Health, Deakin University, Geelong, Victoria Australia

**Keywords:** Bovine lactoferrin (bLf), Apoptosis, Breast cancer, Survivin, p53, Invasion

## Abstract

**Background:**

Iron binding, naturally occurring protein bovine lactoferrin (bLf) has attracted attention as a safe anti-cancer agent capable of inducing apoptosis. Naturally, bLf exists partially saturated (15-20%) with Fe^3+^ however, it has been demonstrated that manipulating the saturation state can enhance bLf’s anti-cancer activities.

**Methods:**

Apo-bLf (Fe^3+^ free) and Fe-bLf (>90% Fe^3+^ Saturated) were therefore, tested in MDA-MB-231 and MCF-7 human breast cancer cells in terms of cytotoxicity, proliferation, migration and invasion. Annexin-V Fluos staining was also employed in addition to apoptotic protein arrays and Western blotting to determine the specific mechanism of bLf-induced apoptosis with a key focus on p53 and inhibitor of apoptosis proteins (IAP), specifically survivin.

**Results:**

Apo-bLf induced significantly greater cytotoxicity and reduction in cell proliferation in both cancer cells showing a time and dose dependent effect. Importantly, no cytotoxicity was detected in normal MCF-10-2A cells. Both forms of bLf significantly reduced cell invasion in cancer cells. Key apoptotic molecules including p53, Bcl-2 family proteins, IAP members and their inhibitors were significantly modulated by both forms of bLf, though differentially in each cell line. Most interestingly, both Apo-bLf and Fe-bLf completely inhibited the expression of survivin protein (key IAP), after 48 h at 30 and 40 nM in cancer cells.

**Conclusions:**

The capacity of these forms of bLf to target survivin expression and modulation of apoptosis demonstrates an exciting potential for bLf as an anti-cancer therapeutic in the existing void of survivin inhibitors, with a lack of successful inhibitors in the clinical management of cancer.

## Background

Apoptosis is a key target for anti-cancer therapy given that it is commonly disrupted in tumourigenesis, allowing diseased cancerous cells to remain and proliferate within the body. Inhibitor of apoptosis proteins (IAPs), play a key role in the ability of cancer to avoid apoptotic signals. Survivin is a 16 kDa protein of the IAP family that enhances cell survival and is over expressed in many cancer cells [1]. Due to its overexpression in cancer, survivin is a key target for cancer therapy. Survivin inhibits apoptosis by directly binding to pro-apoptotic molecules, including caspase family proteins, allowing the progression of cell growth and survival [1–3]. The caspase family consists of initiator caspases-8 and −9 of the extrinsic and intrinsic pathways respectively, as well as executioner caspases-3 and −7.

Lactoferrin (Lf) is a naturally occurring, ferric (Fe^3+^) iron binding glycoprotein [4]. Naturally, Lf has antibacterial and antimicrobial properties and is present in external mammalian excretions including tears, sweat and most importantly milk [4–6]. Bovine lactoferrin (bLf) has been associated with immune boosting and anti-carcinogenic properties [7]. Purified bLf has been shown to increase interferon-γ (IFN-γ), caspase-1 and interleukin-18 (IL-18) [5, 8] as well as increasing the levels of immune cells in various mice models [5, 8, 9]. BLf is approved by the Therapeutic Goods Administration (Australia), Food and Drug Administration (USA) and the European Food Safety Authority for use in food, sports medicine and nutritional products [10–12].

Native bLf (15-20% iron saturated) has been shown to inhibit growth of colon, lung, bladder, squamous and mammary gland tumour cells [13–19] and had been reported to induce apoptosis in colon, lung, stomach and breast cancers [6, 20]. Specifically, apoptosis was reported in two breast cancer cells lines T47D and HS578D with native bLf at concentrations of 0.125-125 μM along with a reduction in cell migration [21]. Activation of apoptosis by bLf has been reported via the modulation of both the extrinsic and intrinsic pathways. BLf has been reported to up-regulate the sensitivity of extrinsic pathway death receptor Fas as well as inducing caspase-3 and −8 cleavage in colon cancer models [22, 23] Results from the same studies also indicated up-regulation of pro-apoptotic Bcl-2 family genes Bax and Bid. Studies have also reported that native bLf significantly decreased the levels of intrinsic anti-apoptotic protein Bcl-2 in stomach cancer cells [20].

Whilst native bLf is approximately 15-20% saturated, it can be modified to forms with different levels of iron: Apo-bLf (<5% iron saturation) and Holo-bLf (100% iron saturated) [4, 24]. In comparison to Apo-bLf, iron saturated bLf is more resistant to *in vivo* gut digestion [25, 26]. The concept that bLf iron saturation levels can affect its anti-tumour activity was not tested until 2008, when we reported that iron saturated (>98%) Fe-bLf, when fed orally to mice, displays anti-tumour properties, increasing apoptosis and cytotoxicity as well as targeting angiogenesis [24]. Importantly, Fe-bLf has been shown to restore red and white bloods cells following chemotherapy [24] and increase the sensitivity of tumours to chemotherapeutic drugs [25, 27]. Fe-bLf encapsulated in ceramic nanocarriers has also proved very effective. When fed orally, we observed a complete inhibition of tumourigenesis in colon cancer xenograft mice in both prevention and treatment models with tumour rejection and regression respectively [25]. In addition, iron-free (Apo-bLf) and selenium saturated bLf (Se-bLf) have shown anti-oxidant effects in colon cancer cells [28, 29].

BLf has a proven high safety profile reported widely by pre-clinical animal studies and human clinical trials [6, 25, 30, 31]. Apo-bLf is thought to have enhanced anti-cancer properties through its capacity to bind free Fe^3+^, acting as an iron chelating agent [32–36]. This could be of particular importance in breast cancer in relation to iron metabolism [37]. Abnormalities in iron metabolism have been associated with chemoresistance in breast cancer cells [38]. As iron is essential for many cellular processes and with a rapid growth rate, high iron levels are essential in the tumour microenvironment [39, 40] allowing for cell growth, proliferation and angiogenesis. Thus chelating agents that can inhibit these processes have great potential.

While apoptosis has been observed in many cancer cell types with native bLf, the specific mechanism of apoptosis in breast cancer cells following Apo-bLf and Fe-bLf treatment remains unclear; we hypothesised that apoptosis would also be initiated in two breast cancer cells MDA-MB-231 and MCF-7 with Apo-bLf and Fe-bLf. It was hypothesised that these two bLf forms would differentially (due to differences in iron level) modulate the apoptosis, and molecules from each of the IAP and caspase families. The effects of both Apo-bLf and Fe-bLf in MDA-MB-231 and MCF-7 human breast adenocarcinoma cell lines were therefore investigated. Both forms of bLf were also tested on non-tumourigenic mammary epithelial cell line, MCF-10-2A. Furthermore, tumourigenic properties such as migration and invasion were also studied in breast cancer cells. Full investigation into the mechanisms and pathways activated in terms of apoptosis following Apo-bLf and Fe-bLf treatment was performed.

## Methods

### Cell culture/Maintenance

MDA-MB-231, MCF-7 and MCF-10-2A cell lines were obtained from the American Type Culture Collection (ATCC). MDA-MB-231 were maintained in Leibovitz’s L15 medium (Life Technologies) supplemented with 10% FBS and antibiotic-antimytotic. MCF-7 cells were maintained in Eagle’s Minimum Essential Medium (EMEM, Life Technologies) supplemented with 10% FBS, antibiotic-antimytotic and 0.01 mg/ml bovine insulin (Life Technologies). MCF-10-2A cells were maintained in Dulbeccos modified Eagle medium (DMEM)/Ham’s F12 medium (Life Technologies) with 10% horse serum, 0.01 mg/ml bovine insulin, 20 ng/ml epidermal growth factor, 100 ng/ml Cholera toxin, 500 ng/ml hydrocortisone and antibiotic-antimytotic. Cells were incubated at 37 °C, MCF-7 and MCF-10-2A cells in the presence of 5% CO_2_.

### Lactoferrin preparation

Apo-bLf (iron free) was prepared from commercial grade pure, endotoxin (LPS) free, native bLf. Briefly, 80 mg/ml native bLf was dissolved in Milli-Q water and iron released by reducing pH to 2.06. The bLf solution was then dialysed in 50 kDa molecular weight cut-off dialysis tubing against 0.1 M citric acid for 48 h and pH adjusted back to 8.0. Fe-bLf (iron-saturated) was prepared by the addition of ferric nitriloacetate (Fe-NTA) to Apo-bLf drop wise until the solution reached a deep red colour indication iron saturation. The Fe-bLf solution was then dialysed against Milli-Q water for 48 h. Protein estimation was performed using the Coomassie Plus (Bradford) Protein Assay (Thermo Scientific) and purification was confirmed via SDS-PAGE (results not shown).

### Lactate dehydrogenase release (LDH) release assay

Cell cytotoxicity was determined using a cytotoxicity detection, lactate dehydrogenase release (LDH) assay kit (Roche). Cells were grown in 96-well plates (2 × 10^5^ cells/ml, 200 μl/well) for 48 h prior to 24 and 48 h treatment with 5, 10, 20, 30 and 40 nM Apo-bLf and Fe-bLf. Supernatants from cells were incubated with LDH reaction mixture and incubated for 30 min in fresh microplates. Plates were then read at a wavelength of 492 nm with a reference of 620 nm. High (100% cytotoxicity) and low (0% cytotoxicity) controls were used to calculate relative percentages of each treatment group.

### CyQUANT® assay

The CyQUANT® cell proliferation assay (Life Technologies) was performed on both MDA-MB-231 and MCF-7 treated cells as per manufacturer’s instructions. Briefly, cells were plated in 96-well plates, treated with both Apo-bLf and Fe-bLf for 24 and 48 h at concentrations of 20, 30 and 40 nM. Treatment media was then removed and mircoplates were frozen at −80 °C overnight. Plates were then thawed and cells were incubated with CyQUANT® GR dye/cell lysis buffer prepared as per kit instructions. Plates were then read at 480 nm excitation and 520 nm emission maxima using a fluorescent spectrophotometer.

### Immunofluorescence

Immunofluorescence was performed on both cancer cells for bLf to determine the localisation of bLf into the cells. Cells were seeded onto 8-chamber well slides and let to grow until 70% confluent. Cells were then treated with either form of bLf at 40 nM for 4 h. Cells were then fixed in 4% paraformaldehyde and blocked in 2% BSA followed by incubation in anti-bovine lactoferrin primary antibody (Bethyl Laboratories) and Alexa-Fluor 594 conjugated anti-goat secondary antibody (Life Technologies) for 1 h each. Cells were then washed in phosphate buffered saline (PBS) before chamber removal and mounting media containing DAPI application. Slides were then covered, sealed and imaged via confocal microscope and software (Leica TCS SP5).

### Migration/Invasion assay

Both migration and invasion assays were performed in 24 well plates with 8 μm pore membrane, ThinCert™ cell culture inserts (Greiner). Briefly, plates were set up with media containing 10% FBS in the lower compartment. Inserts were then placed into wells and 200 μl of 1% FBS media containing 2 × 10^4^ cells was seeded into each insert. To this, treatments of 5 or 10 nM Apo-bLf or Fe-bLf were added and total system was incubated for 24 h. After which, ThinCert^TM^ chambers were removed and membranes fixed in 4% paraformaldehyde. Cells were stinaed with 0.2% crystal violet and cells on the bottom (i.e. those that had migrated through the membrane) were counted (5 representative fields) and averaged. Invasion assays were performed in the same way however an artificial extracellular matrix (ECM) was placed on the membrane prior to cell seeding to test invasive properties. MaxGel™ ECM (Sigma-Aldrich) was used according to manufacturer’s instructions. Both invasion and migration were represented as a percentage of control (untreated) cell counts.

### Annexin-V-fluos apoptosis assay

Apoptosis was detected via an Annexin-V-Fluos staining kit (Roche) according to manufacturer’s protocol. MDA-MB-231 and MCF-7 cells grown in 12 well plates were treated with either Apo-bLf or Fe-bLf at 20 or 40 nM for 24 h. Following treatment, cells were trypsinised and pelleted, washed in PBS and again pelleted. Cells were resuspended in Annexin-V-Fluos labelling solution consisting of Annexin-V-Fluorescein labelling reagent and propidium iodide (PI). Cells were incubated in the labelling solution for 20 min at room temperature before analysis via flow cytometry (BD Biosciences). Cells were gated according to PI and Annexin-V staining. Cells positive for Annexin-V only were considered early apoptotic, cells positive for PI only were considered necrotic and cells double positive were taken as late apoptotic/dead. Assays were performed in duplicate.

### Apoptotic array

Human apoptosis protein arrays (R&D Systems) were employed to screen 35 pro and anti-apoptotic molecules from both the extrinsic and intrinsic pathways. Capture and control antibodies were spotted in duplicate onto nitrocellulose membranes which were subsequently incubated with 250 μg whole cell lysate overnight. Following incubation with kit buffers and reagents, membranes were viewed using chemiluminescence detection and spot densities analysed via Image J (National Institutes of Health).

### Western blotting

Western Blotting was performed with cell lysates prepared from cells treated for 48 h in either Apo or Fe-bLf at concentrations of 20, 30 and 40 nM as well as from untreated cells. After treatments, cells were washed in sterile PBS and lysed in RIPA buffer (0.6057 g Tris base, 0.877 g NaCl, 10 ml Nonident P‐40, 5 ml 10% Na‐deoxycholate, 1 ml 10% sodium dodecyl sulphate, pH set to 7.5, adjusted to 100 ml with H_2_O) for 30 min before centrifugation and supernatant collection. 100 μg of total protein (determined via Bradford assay) was then used for sodium dodecyl sulphate polyacrylamide gel electrophoresis (SDS-PAGE). Following electrophoresis, proteins were transferred to PVDF membranes which were subsequently blocked in 1% skim milk powder made up in Tris buffered saline (TBS, 2.42 g Tris base, 8 g NaCl, made up to 1 L with autoclaved Milli‐Q water, pH 7.6) for 1 h. Membranes were then incubated overnight in appropriate primary antibodies (Survivin and Tubulin: Santa Cruz, all other antibodies: Cell Signalling) diluted to 1:1000 in TBS followed by 1 h in appropriate secondary antibodies (Sigma-Aldrich) with washes after each incubation. Membranes were exposed to chemiluminescent reagents and imaged via ChemiDoc XRS camera (BioRad). Band densities analysed via Image J (National Institutes of Health).

### Statistical analysis

All statistical analysis was performed via Student’s t-test.

## Results

### BLf induces cytotoxicity and reduces the proliferative ability of MDA-MB-231 and MCF-7 cells

The effect of both Apo-bLf and Fe-bLf on cytotoxicity and proliferation were evaluated in the breast cancer cells MDA-MB-231 and MCF-7. Cells were treated with bLf for 24 and 48 h at concentrations of 0, 5, 10, 20, 30 and 40 nM. In order to quantify cell death, Lactate dehydrogenase (LDH) cytotoxicity assays were employed to determine cytotoxicity.

**Fig. 1:**
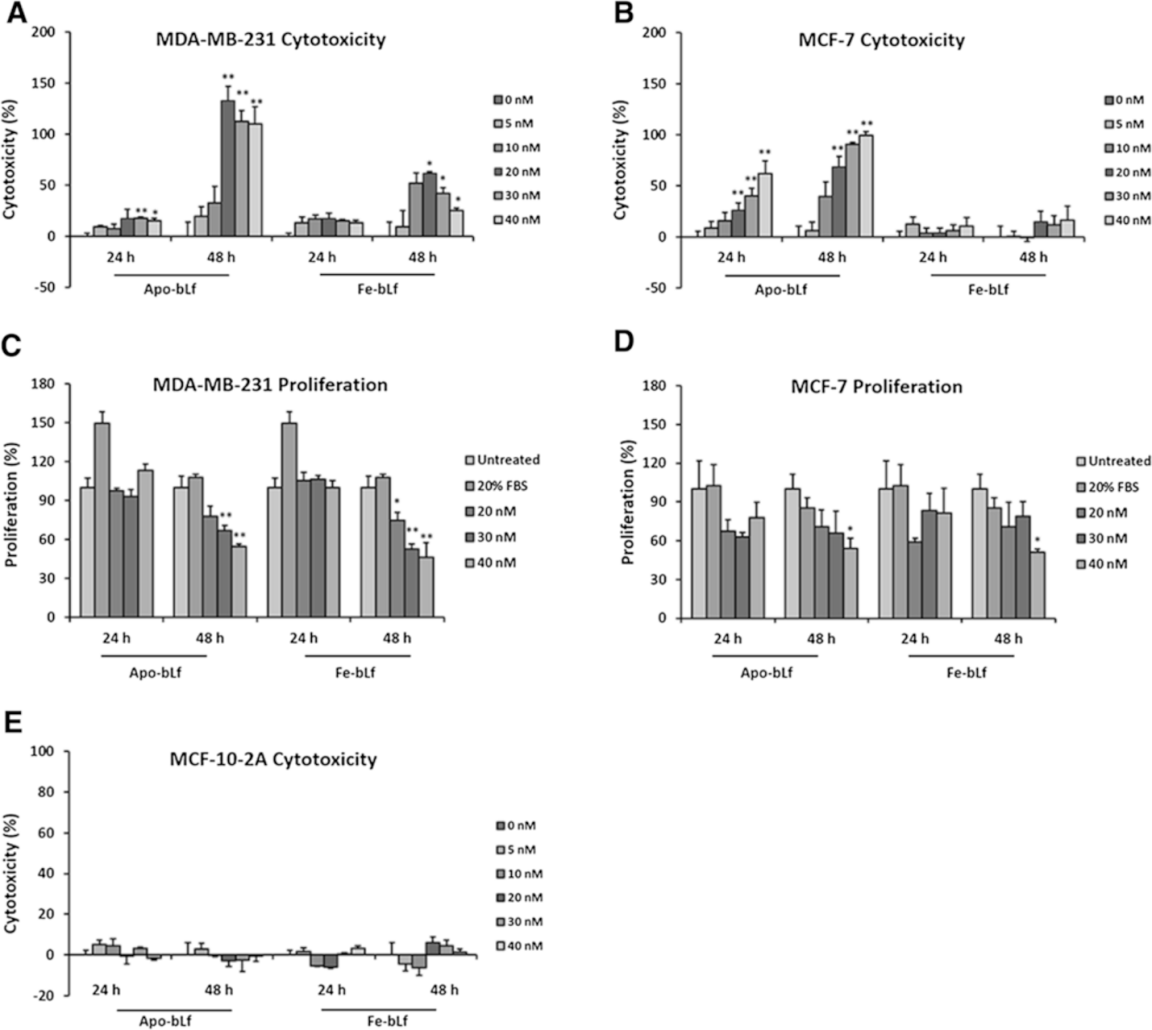
Cytotoxicity and proliferation in MDA-MB-231, MCF-7 and MCF-10-2A cells following Apo-bLf and Fe-bLf treatments. Lactate dehydrogenase assay (LDH) results demonstrating cytotoxicity in cells after 24 and 48 h Apo and Fe-bLf treatments in MDA-MB-231 (**a**), MCF-7 (**b**) and MCF-10-2A cells (**e**). CyQUANT® assay results represent cell proliferation levels after 24 and 48 h Apo and Fe-bLf treatments in MDA-MB-231 (**c**) and MCF-7 cells (**d**) including high (20% FBS) and low (untreated) controls. Data represented as mean with + SEM (n = 6). * = *p* < 0.05 compared with untreated (0 nM) group, ** = *p* < 0.01 compared with untreated (0 nM). Statistical analysis performed using Student’s t-test

Apo-bLf was the most effective at inducing cell cytotoxicity with significant increases in cytotoxicity at concentrations of 20, 30 and 40 nM in MDA-MB-231 (133.05%, *p* = 0.003, 112.53%, *p* = 0.003 and 110.17%, *p* = 0.007, respectively) and MCF-7 cells (68.58%, *p* = 0.001, 90.68%, *p* = 0.00001 and 99.31%, *p* = 0.000006, respectively) after 48 h (Fig. 1a). Furthermore, Apo-bLf demonstrated significant toxicity after 24 h in MCF-7 cells at concentrations of 20 nM (25.93%, *p* = 0.02), 30 nM (40.09%, *p* = 0.001) and 40 nM (61.97%, *p* = 0.001) (Fig. 1b). Fe-bLf demonstrated significant increases in cytotoxicity in MDA-MB-231 cells at concentrations of 10, 20 and 30 nM (51.81%, *p* = 0.04, 61.76%, *p* = 0.01 and 42.01%, *p* = 0.048) after 48 h (Fig. 1a) yet no significant cytotoxicity in MCF-7 cells (Fig. 1b).

CyQUANT® assay results indicated a reduction in proliferation following treatments with both Apo-bLf and Fe-bLf after 48 h in MDA-MB-231 cells (Fig. 1c). Apo-bLf significantly reduced proliferation at concentrations of 30 and 40 nM (67.30%, *p* = 0.01 and 54.87%, *p* = 0.003, respectively) and Fe-bLf at concentrations of 20, 30 and 40 nM (74.86%, *p* = 0.04, 52.89%, *p* = 0.003 and 46.40%, *p* = 0.008) after 48 h. Furthermore, Apo and Fe-bLf reduced proliferation after 48 h in MCF-7 cells at 40 nM (53.93%, *p* = 0.03 and 51.35%, *p* = 0.01 respectively) (Fig. 1d). No significant increase in proliferation was observed in any treatment condition in either cell line.

**Fig. 2:**
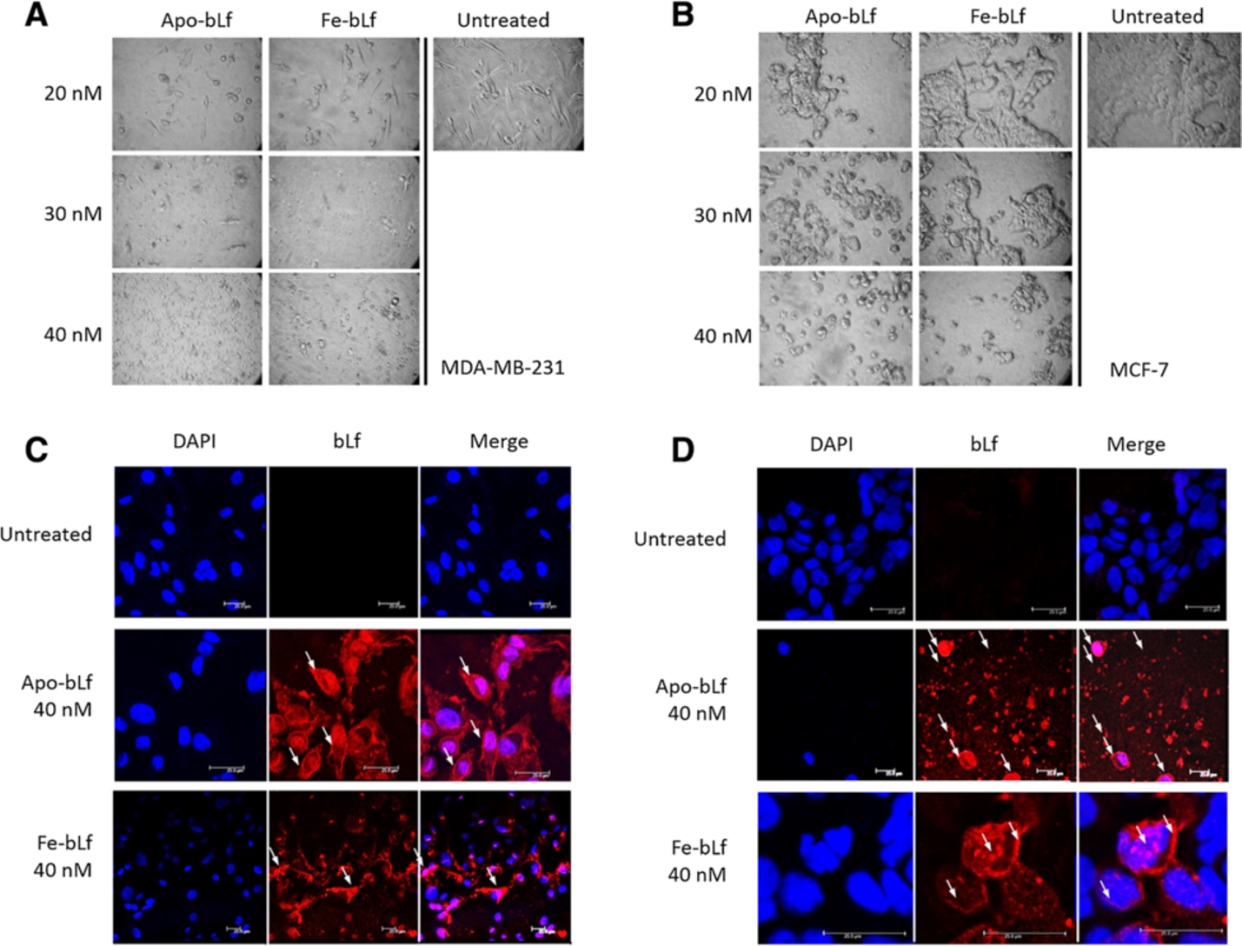
Cell morphology and internalization of bLf in MDA-MB-231 and MCF-7 cells. **a** MDA-MB-231 cells after 48 h treatments with both Apo-bLf and Fe-bLf at concentrations of 20, 30 and 40 nM as well as untreated cells. **b** MCF-7 cells after 48 h treatments with both Apo-bLf and Fe-bLf at concentrations of 20, 30 and 40 nM as well as untreated cells. All images at 400X magnification. Confocal microscope images displaying internalisation of 40 nM Apo-bLf and Fe-bLf in both MDA-MB-231 (**c**) and MCF-7 (**d**) cells after 4 h. Arrows indicate regions on the membrane, cytoplasm and nucleus where bLf has internalised. Cells stained with DAPI nuclear stain (blue) and immunolabelled with anti-bovine lactoferrin antibody followed by Alexa-Fluor TRITC conjugated secondary antibody (red). Scale bars indicate 25 μm

Morphological images (Fig. 2a and b) indicated significant cell death after 48 h. Cells in treatment groups showed detachment from culture surfaces, blebbing and rounded edges. Cell fragmentation and decrease in number was also observed compared with untreated cells over the same time period. These effects were observed more frequently with increasing concentration in MDA-MB-231 (Fig. 2a) and MCF-7 (Fig. 2b). These results indicate a time and dose dependant effect of Apo-bLf on MDA-MB-231 and MCF-7 cells in terms of increasing cell cytotoxicity and decreasing cell proliferation. Furthermore, these results also highlight that Fe-bLf is less effective when compared with Apo-bLf in these breast cancer cells.

### Apo-bLf and Fe-bLf show no cytotoxic effects in MCF-10-2A mammary epithelial cells

Both Apo-bLf and Fe-bLf were also tested in a normal, non-tumourigenic mammary epithelial cell line MCF-10-2A. LDH cytotoxicity assays were performed on cells treated for 24 and 48 h at concentrations of 5, 10, 20, 30 and 40 nM. No significant increase in cytotoxicity was observed after either 24 or 48 h with any of the Apo-bLf and Fe-bLf treatments (Fig. 1e).

### Apo-bLf and Fe-bLf efficiently internalizes into treated cells

BLf is known to internalize into normal and cancer cells through cell surface (membrane) receptor mediated endocytosis [10, 30, 41, 42]. In order to determine the level of internalisation and localisation of bLf within breast cancer cells, we performed immunofluorescence. Cells were treated with either Apo-bLf or Fe-bLf at 40 nM for 4 h. A reduced time period was used to reduce cell death so that cells could still be visualised by confocal microscopy. Both forms of bLf internalised into MDA-MB-231 (Fig. 2c) cells with fragmentation displayed in cells treated with Fe‐bLf at 40 nM. Both forms of bLf were localised in the membrane, cytoplasm and nucleus of the cells. Untreated MCF-7 cells displayed healthy, cluster formation (Fig. 2d). After treatment with both Apo-bLf and Fe-bLf, internalisation of bLf into the membrane, cytoplasm and nucleus was observed (Fig. 2d). It is apparent that Apo-bLf induces mass cell fragmentation (Fig. 2d) which is due to the high cytotoxic potential of Apo-bLf. Cell clusters were also much smaller in treated cells compared with untreated cells, indicating the effect of treatment on cell viability following bLf internalisation.

### Apo-bLf and Fe-bLf treatments decrease the migration and invasion potential of breast cancer cells

**Fig. 3:**
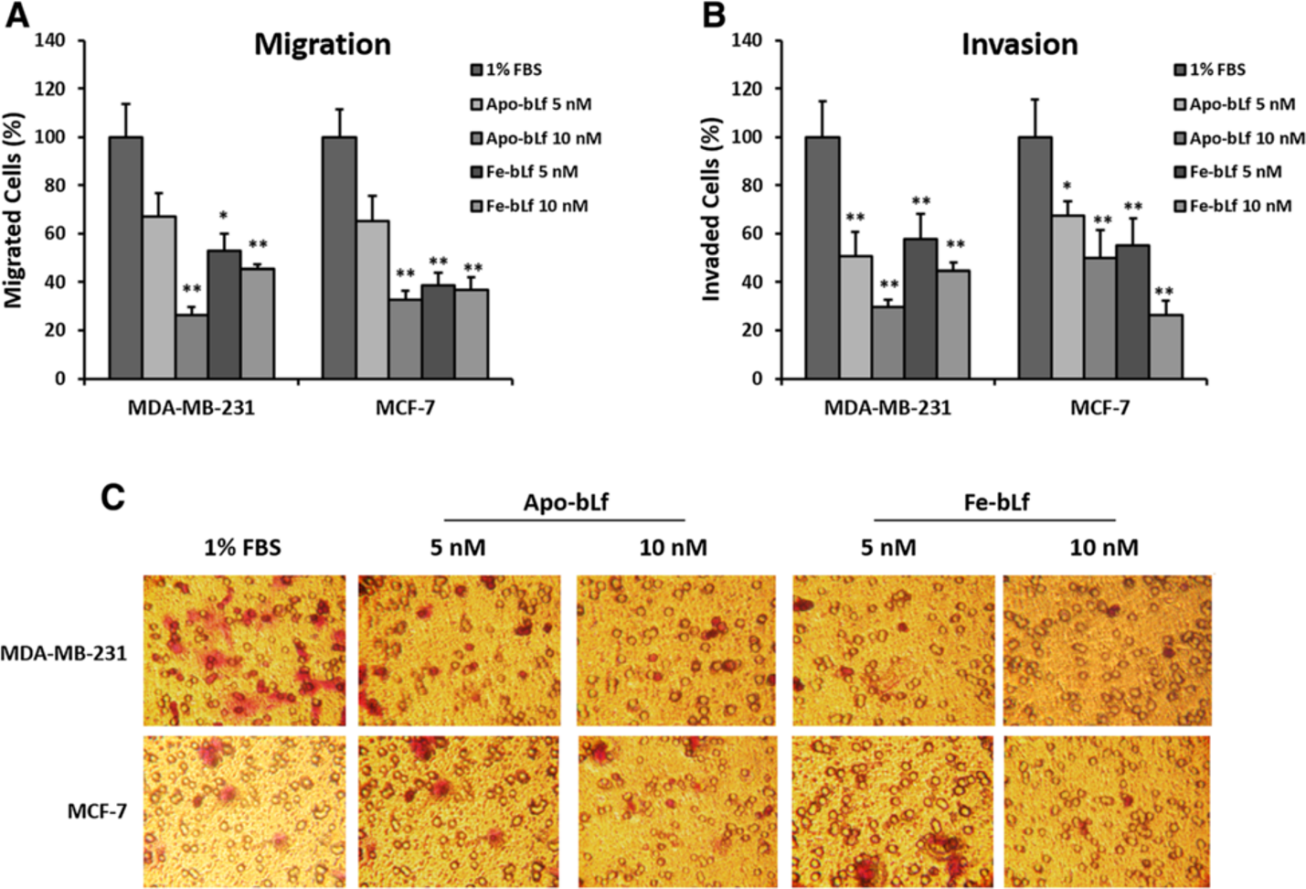
Effect on migration and invasion capacity of MDA-MB-231 and MCF-7 cells after treatment with bLf. Migration of MDA-MB-231 and MCF-7 (**a**) cells after bLf treatments for 24 h represented as a percentage of untreated (1% FBS) control migration. Invasion of MDA-MB-231 and MCF-7 cells (**b**) after bLf treatments for 24 h represented as a percentage of untreated (1% FBS) control invasion. * = *p* < 0.05 compared with 1% FBS group. Representative images (250X magnification) of invaded cells stained with crystal violet (**c**)

As migration and invasion are key properties of cancer cells leading to metastases and secondary tumour sites within the body, assays were performed to determine the effects of bLf in regards to these properties. Migration assays indicated a significant reduction in the capacity of MDA-MB-231 and MCF-7 (Fig. 3a) cells to migrate through a porous membrane after treatment for 24 h with each Apo-bLf and Fe-bLf at concentrations of 5 and 10 nM. The greatest reduction in migration was observed with 10 nM Apo-bLf with 26.45% (*p* = 0.001) of MDA-MB-231 and 38.78% (*p* = 0.001) of MCF-7 cells migrating. Invasion assays (Fig. 3b and c) were performed with ECM. The same trend was observed in MDA-MB-231 cells with 29.85% (*p* = 0.001) of cells invading after treatment with 10 nM Apo-bLf. MCF-7 showed a large reduction with 10 nM Apo-bLf (50.00%, *p* = 0.003) however the greatest reduction in invasion was with 10 nM Fe-bLf treatments with 26.25% (*p* = 0.00008) invasion.

### Both bLf forms induce significant apoptosis at a different rates in MDA-MB-231 and MCF-7 cells

**Fig. 4:**
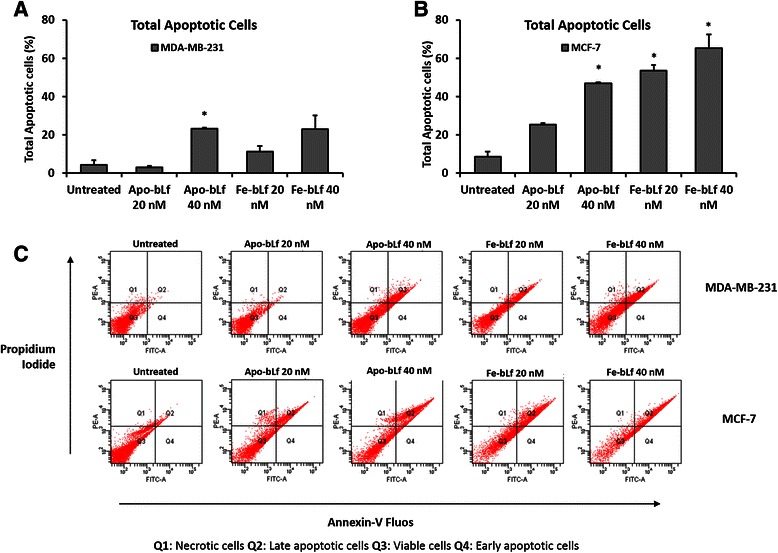
BLf-induced apoptosis in MDA-MB-231 and MCF-7 cells. Annexin-V-Fluos staining detected apoptosis in MDA-MB-231 and MCF-7 cells following bLf treatments. Cells were treated for 24 h with Apo-bLf and Fe-bLf at concentrations of 20 nM and 40 nM. **a** Total apoptotic MDA-MB-231 and **b** MCF-7 cells following bLf treatments. Flow cytometry plots of MDA-MB-231 and MCF-7 cells (**c**). Q1: Necrotic cells, Q2: Late apoptotic/Dead cells, Q3: Viable cells, Q4: Early apoptotic cells. Total apoptotic cells were the sum of early and late apoptotic cell populations. Data represented as means +SEM, student t-test used for statistical analysis

In order to determine if cell death was caused by apoptosis, Annexin-V-Fluos labelling was performed on each MDA-MB-231 and MCF-7 cells (Fig. 4). Cell were treated with Apo-bLf or Fe-bLf at 20 and 40 nM for 24 h. Labelling was detected via Flow cytometry and gating was performed on both dual (Fig. 4c) and single channel plots (Annexin-V and Propidium Iodide). Results indicated increases in apoptotic cell death in MDA-MB-231 cells treated with bLf with significant increased total apoptosis in cells treated with Apo-bLf at 40 nM (23.30%, p = 0.03) (Fig. 4a). Results from MCF-7 cells showed increased apoptosis in all treatment groups with significant apoptosis observed in cells treated with 40 nM Apo-bLf (46.85%, *p* = 0.05) and Fe-bLf at 20 nM (53.60% *p* = 0.02) and 40 nM (65.15%, *p* = 0.04) (Fig. 4b). Furthermore, decreased viable cells were observed in MDA-MB-231 cells after Apo-bLf at 40 nM and in both Fe-bLf treatment groups and in all MCF-7 treatment groups however no significant increase in necrotic cells was observed in any of the treatment groups (Table 1). This indicated that both Apo-bLf and Fe-bLf were inducing apoptosis in each cell line.

**Table 1:** Annexin-V results following bLf treatments in breast cancer cells

		*Early Apoptotic Cells*	*Late Apoptotic Cells*	*Viable Cells*	*Necrotic Cells*
*MDA-MB-231*	*Untreated*	1.75 ± 1.15	2.50 ± 2.10	94.85 ± 3.25	0.85 ± 0.05
	*Apo-bLf 20 nM*	1.90 ± 0.60	1.10 ± 0.40	96.2 ± 1.40	0.85 ± 0.35
	*Apo-bLf 40 nM*	5.55 ± 0.65	17.75 ± 0.05*	75.4 ± 0.10*	1.10 ± 0.5
	*Fe-bLf 20 nM*	1.90 ± 0.30	9.30 ± 3.30	87.7 ± 3.40	1.10 ± 0.20
	*Fe-bLf 40 nM*	3.75 ± 1.95	19.35 ± 6.85	74.00 ± 8.80	2.90 ± 0.00
*MCF-7*	*Untreated*	3.40 ± 3.20	5.25 ± 3.25	88.60 ± 7.30	2.70 ± 0.90
	*Apo-bLf 20 nM*	2.50 ± 0.40	22.85 ± 9.65	64.95 ± 3.85	9.75 ± 6.15
	*Apo-bLf 40 nM*	6.15 ± 0.55	40.7 ± 7.30*	51.00 ± 6.70*	2.20 ± 0.00
	*Fe-bLf 20 nM*	12.30 ± 0.30	41.3 ± 1.40*	45.45 ± 1.65*	0.95 ± 0.05
	*Fe-bLf 40 nM*	21.65 ± 1.65*	43.5 ± 12.10	34.15 ± 10.05*	0.70 ± 0.40

Annexin-V-Fluos staining results determined via Flow cytometry. Total populations acquired were plotted in terms of both single and dual fluorophores. Dual channel plots were divided in to quadrants: Q1: Necrotic cells, Q2: Late apoptotic/Dead cells, Q3: Viable cells, Q4: Early apoptotic cells. Single channel plots were used to determine total numbers of cells labelled with Annexin-V or propidium iodide. Table 1 shows average values for individual gating of both dual and single channel plots + SEM. Statistical analysis was performed via Student’s t-test

**Fig. 5:**
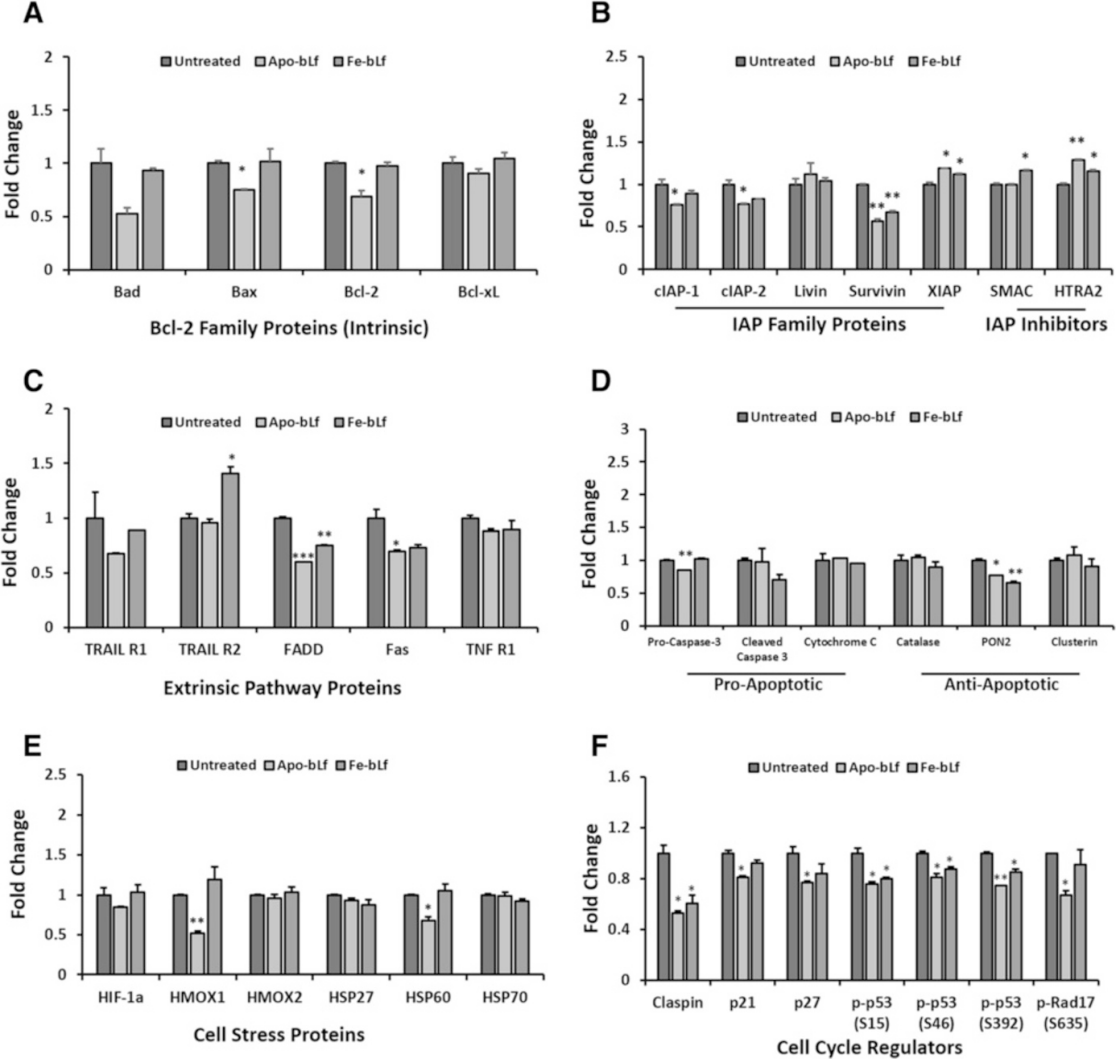
Apoptosis protein array MDA-MB-231 breast cancer cells. Apoptosis protein array results following incubation of MDA-MB-231 cell lysate after treatments with Apo-bLf and Fe-bLf at 40 nM for 24 h. Cell lysate (250 μg) incubated with nitrocellulose membrane pre-labelled with capture antibodies (duplicate spots) and detected via chemiluminescence. **a** Bcl-2 family proteins Bad, Bax, Bcl-2 and Bcl-xL. **b** Inhibitor of apoptosis (IAP) proteins cIAP-1 and 2, Livin, Survivin and XIAP, and inhibitors SMAC and HTRA2. **c** Extrinsic pathway proteins and receptors TRAIL 1 and 2, FADD, Fas and TNF R1. **d** Pro-apoptotic proteins Pro-caspase-3, Cleaved Caspase-3 and Cytochrome C, anti-apoptotic proteins Catalase, PON2 and Clusterin. **e** Cell stress proteins HIF-1α, HMOX1, HMOX2, HSP27, HSP60 and HSP70. **f** Claspin, p21, p27, phospho-p53 (S15), phospho-p53 (S46), phospho-p53 (S392) and phospho-Rap17 (S635). Average density determined using ImageJ software and fold change calculated compared with untreated. Data represented as means with + SEM. * = *p* < 0.05, ** = *p* < 0.01, *** = *p* < 0.001 compared with the untreated group. Statistical analysis was performed via Student’s t-test

**Fig. 6:**
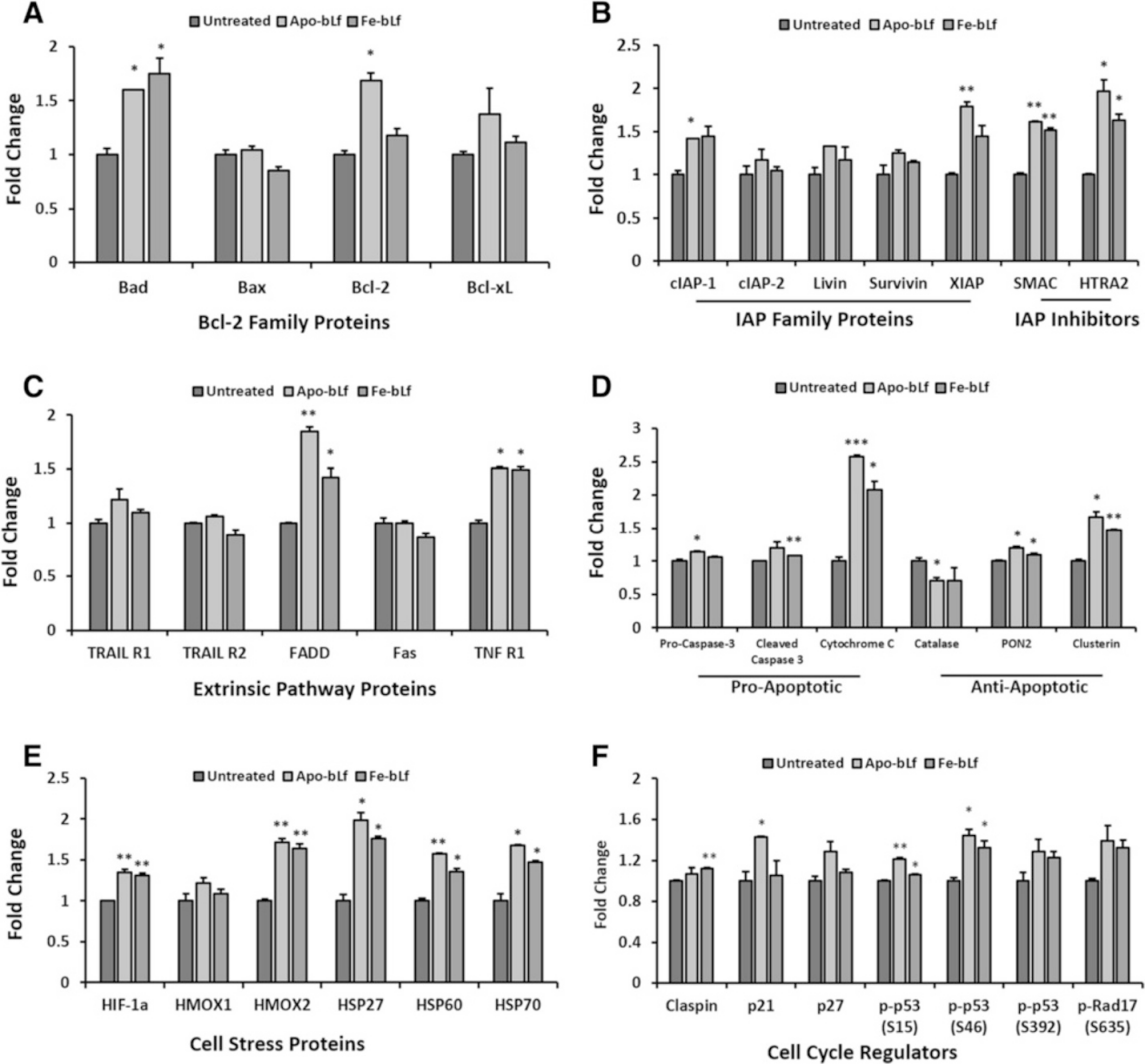
Apoptosis protein array MCF-7 breast cancer cells. Apoptosis protein array results following incubation of MCF-7 cell lysate after treatments with Apo-bLf and Fe-bLf at 40 nM for 24 h. Cell lysate (250 μg) incubated with nitrocellulose membrane pre-labelled with capture antibodies (duplicate spots) and detected via chemiluminescence. **a** Bcl-2 family proteins Bad, Bax, Bcl-2 and Bcl-xL. **b** Inhibitor of apoptosis (IAP) proteins cIAP-1 and 2, Livin, Survivin and XIAP, and inhibitors SMAC and HTRA2. **c** Extrinsic pathway proteins and receptors TRAIL 1 and 2, FADD, Fas and TNF R1. **d** Pro-apoptotic proteins Pro-caspase-3, Cleaved Caspase-3 and Cytochrome C, anti-apoptotic proteins Catalase, PON2 and Clusterin. **e** Cell stress proteins HIF-1α, HMOX1, HMOX2, HSP27, HSP60 and HSP70. **f** Claspin, p21, p27, phospho-p53 (S15), phospho-p53 (S46), phospho-p53 (S392) and phospho-Rap17 (S635). Average density determined using ImageJ software and fold change calculated compared with untreated. Data represented as means with + SEM. * = *p* < 0.05, ** = *p* < 0.01, *** = *p* < 0.001 compared with untreated group. Statistical analysis was performed via Student’s t-test

To determine the mechanism of action of apoptosis induced by bLf, apoptotic protein arrays were performed on each MDA-MB-231 (Fig. 5) and MCF-7 (Fig. 6) cells following treatments with Apo-bLf and Fe-bLf for 24 h. Results indicated different mechanisms of action between the two forms of bLf and between the cells. MDA-MB-231 showed significant reduction in survivin expression along with increased expression of HTRA2 with each Apo-bLf and Fe-bLf (Fig. 5b). In addition, Apo-bLf reduced cIAP2 and SMAC was increased with Fe-bLf (Fig. 5b). In MCF-7 cells, HTRA2 and SMAC were both significantly increased with both forms of bLf, with a stronger effect observed with Apo-bLf (Fig. 6b). These results indicate that bLf is having an impact on the IAP mechanism, allowing apoptosis to progress by activating SMAC and HTRA2 which subsequently bind IAP proteins, preventing their inhibition of the caspase cascade.

Furthermore, results indicate apoptosis in MDA-MB-231 cells occurring via the intrinsic pathway with a significant reduction in expression of anti-apoptotic proteins in Bcl-2 (Apo-bLf) and PON2 (both forms of bLf) (Fig. 5a). A reduction in extrinsic proteins FADD and Fas (Fig. 5c) was also observed however the opposite occurring in MCF-7 (Fig. 6c) with extrinsic proteins FADD and TNF R1 each upregulated with both forms of bLf. Furthermore, MCF-7 cell also show significant increases of pro-apoptotic proteins Bad and cytochrome C (Fig. 6a & d).

Cell stress proteins in MDA-MB-231 cells, HMOX1 and HSP60 were both reduced with Apo-bLf (Fig. 5e) as were cell cycle regulators claspin, p21, p27, phospho-p53 (S15, S46 and S392) and phospho-Rad17 (S635) (Fig. 5 f). Claspin was also significantly reduced with both Apo-bLf and Fe-bLf (Fig. 5 f). In MCF-7 cells, cell stress proteins were increased by both Apo-bLf and Fe-bLf including HIF1α, HMOX2, HSP27, HSP60 and HSP70 (Fig. 6e). Finally, cell cycle regulator p21 was increased in MCF-7 cells with Apo-bLf, Claspin with Fe-bLf and phospho-p53 S15 and S46 were increased with both Apo-bLf and Fe-bLf (Fig. 6 f).

### Apo-bLf and Fe-bLf forms induce increase in caspase cleavage

Following the observations form the Apoptotic array that bLf modulated aspects of both the intrinsic and extrinsic pathways, as well as increases in cleaved caspase-3, the effect of bLf on cleaved initiator caspase-8 (extrinsic) and caspase-9 (intrinsic) were assed via Western blotting. In addition, pro and cleaved effector caspase-3 were also analysed. MDA-MB-231 and MCF-7 cells were both treated with 40 nM Apo-bLf and Fe-bLf for 24 h. Following treatments, cells were lysed with RIPA buffer and lysates were separated via SDS-PAGE followed by Western blotting. Identical gels were run and Western blotting performed for GAPDH. Fold change was calculated by determining band densities via ImageJ software, normalizing proteins with GAPDH and calculating fold change relative to untreated cells.

**Fig. 7:**
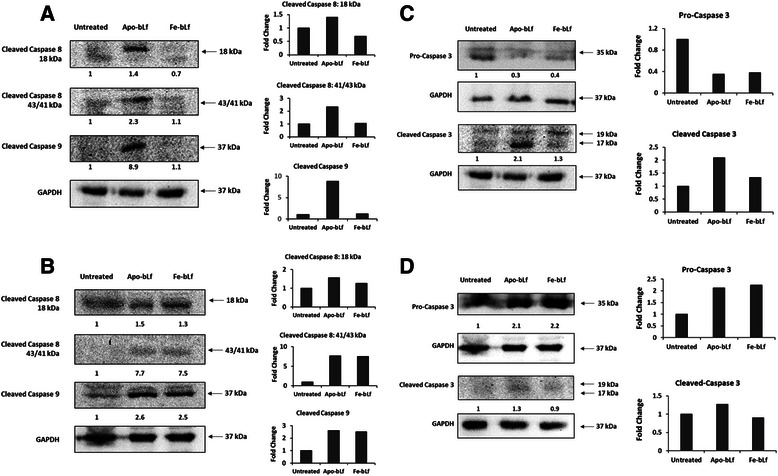
BLf induces caspases-3, −8 and −9 cleavage in breast cancer cell lines. Western blot analysis of cleaved caspases-8 and −9 in MDA-MB-231 (**a**) and MCF-7 (**b**) cells following treatment with 40 nM Apo-bLf and Fe-bLf for 24 h. Western blot analysis of pro and cleaved caspase-3 in MDA-MB-231 (**c**) and MCF-7 (**d**) cells following treatment with 40 nM Apo-bLf and Fe-bLf for 24 h. Lysates were collected and 100 μg loaded and run on standard SDS-PAGE followed by transfer to PVDF membranes. Membranes were then blocked with 1% skim milk followed by incubation with anti-cleaved caspases-3, −8 and −9 primary antibodies (Cell Signalling) and anti-mouse secondary antibody (Sigma-Aldrich). Membranes were viewed via XRS camera. Band densities determined by ImageJ software and compared with untreated (0 nM). Separate, identical gels were run for GAPDH (Santa Cruz) which were used to normalize band densities. Band density analysis was performed using ImageJ software. Fold change was calculated relative to untreated cells. Relative fold change values per band are indicated below blots as well as plotted on graphs

Cleaved caspases-8 and −9 were increased with 40 nM Apo-bLf in MDA-MB-231 cells (Fig. 7a). Fe-bLf however did not show increases in either cleaved caspase-8 or −9. Apo-bLf increased the active fragment (18 kDa) of cleaved caspase-8 by 2.3 fold compared with untreated cells. Most notably however, cleaved caspase-9 was increased by 8.9 fold with Apo-bLf.

In MCF-7 cells, both cleaved caspases-8 and −9 were increased with 40 nM Apo-bLf and Fe-bLf (Fig. 7b). The effects of Apo-bLf and Fe-bLf were similar in their effects on cleaved caspases-8 and −9. Active caspase-8 (18 kDa fragment) was increased to 1.5 and 1.3 fold with Apo-bLf and Fe-bLf respectively while the remaining, unused fragments (41 and 43 kDa) from the pro-caspase was increased to 7.7 and 7.5 fold respectively indicating large levels of cleavage. Furthermore, cleaved caspase-9 was increased by 2.6 and 2.5 fold with Apo-bLf and Fe-bLf respectively. Analysis of effector caspase-3 was then performed in MDA-MB-231 (Fig. 7c) and MCF-7 (Fig. 7d) cells. In MDA-MB-231 cells, a large decrease in pro-caspase-3 was observed with both Apo-bLf and Fe-bLf with expression reduced to 0.3 and 0.4 fold respectively of untreated cells. This was taken to indicate caspase cleavage and this was further confirmed by Western blot analysis of cleaved caspase-3 levels in MDA-MB-231 cells. Cleaved caspase-3 levels increased by 2.1 fold in cells treated with Apo-bLf and slight increase by 1.3 fold in cells treated with Fe-bLf. In MCF-7 cells, pro-caspase-3 increased with both Apo-bLf (2.1 fold) and Fe-bLf (2.2 fold). Cleaved caspase-3 was relatively unchanged in Fe-bLf and a slight increase observed with Apo-bLf (1.3 fold) (Fig. 7d).

### BLf treatment leads to time and dose dependent down-regulation of survivin protein expression in both MDA-MB-231 and MCF-7 cells

**Fig. 8:**
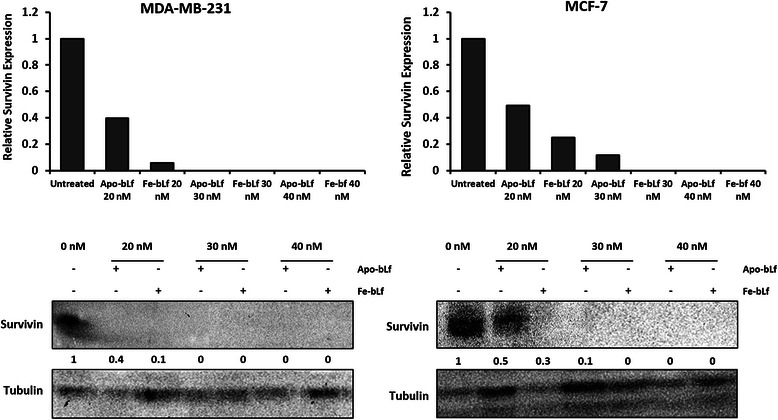
BLf reduces survivin expression in breast cancer cell lines. Western blotting for survivin of MDA-MB-231 and MCF-7 cell lysates following treatments for 48 h with Apo and Fe-bLf at 20, 30 and 40 nM. Lysates were collected and 100 μg loaded and run on standard SDS-PAGE followed by transfer to PVDF membranes. Membranes were then blocked with 1% skim milk followed by incubation with anti-survivin primary antibody (Santa Cruz) and anti-mouse secondary antibody (Sigma-Aldrich). Membranes were viewed via XRS camera. Band densities determined by ImageJ software and compared with untreated (0 nM). Separate, identical gels were run for Tubulin which were used to normalize band densities. Band densities determined by ImageJ software and compared with untreated (0 nM). Separate, identical gels were run for Tubulin (Santa Cruz) which were used to normalize band densities. Band density analysis was performed using ImageJ software. Fold change was calculated relative to untreated cells. Relative fold change values per band are indicated below blots as well as plotted on graphs

As survivin is a key IAP protein in cancer and significant reduction was observed in MDA-MB-231 cells in apoptotic arrays following 24 h treatments (Fig. 5b), Western blotting was performed on a greater concentration range for 48 h in each cell line. Cells were treated with Apo-bLf and Fe-bLf at 20, 30 and 40 nM and cell lysates were blotted for survivin. Survivin was detected in both untreated lysates (Fig. 8). Survivin was greatly reduced in all treatment groups with reduction in MDA-MB-231 cells to 0.4 and 0.1 fold with 20 nM Apo-bLf and Fe-bLf respectively. Survivin expression in MCF-7 cells was reduced to 0. 5 and 0.3 fold with 20 nM Apo-bLf and Fe-bLf and to 0.1 fold that of untreated cells with 30 nM Apo-bLf. No survivin was detected in MDA-MB-231 cells with either form of bLf at concentrations of 30 and 40 nM. Survivin was also not detected in MCF-7 cells after treatment with Fe-bLf at 30 nM and both forms at 40 nM. Western blotting confirmed findings from the apoptotic array and provided important evidence for the mechanism of bLf in terms of its apoptosis-inducing potential.

## Discussion

The presence of cell fragmentation (apoptotic bodies) in the images taken of cells after treatment indicates the strong cytotoxic effects of bLf in breast cancer cells. Increased growth inhibition and apoptosis have been demonstrated in native bLf treated colon, lung and squamous cancer cells *in vivo* [18, 19]. Furthermore, our findings in 2008 demonstrated that mice fed with Fe-bLf showed greater reduction in Lewis lung carcinoma, B16 melanoma and EL4 lymphoma tumour size than identical mice fed with natural bLf or Apo-bLf [24]. Interestingly, in the present study, both human breast carcinoma derived cancer cells showed increased cytotoxicity with Apo-bLf compared with Fe-bLf particularly after 48 h. This enhanced sensitivity of breast cancer cells to Apo-bLf may be tissue or cell line specific, and could possibly be related to iron metabolism in breast cancer cells with Apo-bLf playing a role in chelation and iron sequestering. Fe-bLf however was able to induce higher levels of apoptosis in MCF-7 cells in Annexin-V assays. It is also noteworthy to mention that native bLf has been reported to induce apoptosis and inhibit proliferation in T47D and HS578D breast cancer cell lines [21] however concentrations used were much higher (0.125-125 μM, compared with 5–40 nM in this study). While it is possible that the difference in dosage levels is cell specific, through published [10, 25] and unpublished work in our laboratory, we believe this isn’t the case. We have observed similar cytotoxicity, growth inhibition and apoptosis effects of bLf forms at the nanomolar concentration range, over a range of cell lines obtained from human carcinomas of different origin such as colon (SW480 and Caco-2), liver (Hep3B) and melanoma (SK-MEL-2 and SK-MEL-28). Moreover, The study in in T47D and HS578D breast cancer cell lines [21], was performed with native bLf (15-20% Fe^3+^ saturated), thus the dosage level difference could be due to the modification of iron saturation levels of bLf given that the work we have performed is with Apo-bLf (iron free) and Fe-bLf (iron saturated). As it is vital to keep dose levels as low as possible, it is encouraging that modification of bLf in terms of iron saturation can allow such reduced dosage levels to be achieved. Moreover, due to its gut-enzyme resistant nature leading to increased bioavailability at tumour sites following oral administration [25], Fe-bLf has a better translation potential as a therapeutic. Encouragingly, no cytotoxicity was detected in normal mammary epithelial cells, MCF-10-2A even after 48 h with either form of bLf tested. This is an important finding given the systemic toxicity often associated with traditional chemotherapeutic agents.

Invasion and migration assays indicated that both forms of bLf have the capacity to reduce these metastatic properties of both MDA-MB-231 and MCF-7 cells. Previously, native bLf has been shown to inhibit migration in breast cancer cells via scratch test assays [21]. We report here however that iron free and >90% iron saturated bLf each have the capacity to reduce both migration and invasion in both MDA-MB-231 and MCF-7 cells. It is also prudent to note that Apo-bLf and Fe-bLf were used at concentration of 5 and 10 nM compared with 12.5 μM of native bLf in previous studies. In addition, it was observed that cells in untreated groups were able to fully pass through the pores whereas treated cells showed limited movement and were unable to completely pass through the pores. This possibly indicates that more time would be required for them to translocate and that bLf can reduce their movement capacity. This is an important and novel finding as migration and particularly invasion are key factors of cancer cells allowing them to travel through the body and create secondary cancer sites.

The ability of cancer cells to systematically avoid apoptotic mechanisms and signals is directly linked to their uncontrolled growth and proliferation subsequently allowing invasion and metastasis throughout the body [43]. We report here that both Apo-bLf and Fe-bLf significantly induce apoptosis in MDA-MB-231 and MCF-7 cells in a dose dependent manner. Apoptosis induced by bLf has been reported in several instances via both the extrinsic and intrinsic pathways [7, 20, 22, 23, 44]. Furthermore apoptosis was reported in two human breast cancer cell lines (different to the two cancer cell lines used in the present study) following native bLf treatment [21]. BLf has been shown to up-regulate the sensitivity of extrinsic pathway death receptor Fas in the colon mucosa of azoxymethane-induced colon tumour bearing rats [22] as well as up-regulation of the active forms of both caspase-3 and −8 after treatments with bLf [23]. In addition, studies have also reported that bLf reduces the levels of intrinsic protein Bcl-2 in stomach cancer cells [20] as well as caspase-3 cleavage in squamous cell carcinoma [44]. In line with these findings, we report here that in MDA-MB-231 cells, Bcl-2 expression is reduced with Apo-bLf as is Pro-caspase-3, indicating possible cleavage (Fig 5a & d) which was confirmed via Western blotting (Fig 7c).

Lactoferricin, a peptide derived from lactoferrin has also demonstrated increased apoptosis via the intrinsic pathway in leukemic and breast carcinoma cells by activating caspases-3 and -9 but not caspase-8 [45, 46]. However, it was since shown that lactoferricin induces apoptosis in B-lymphoma in a caspase-independent fashion [47] and shown to induce apoptosis and activate caspases-3, −7, −8 and −9 in gastric cancer thus implying that lactoferricins activity may be tissue/cell specific [48]. This appears to be possible in the case of the current study as MDA-MB-231 and MCF-7 appear to have distinct differences in the apoptotic factors activated following incubation with Apo-bLf and Fe-bLf.

It is apparent that bLf-induced apoptosis is higher in MCF-7 cells as observed through Annexin-V and by the levels of apoptotic molecule modulation determined via apoptotic array including cell stress proteins and cell cycle regulators. It may be possible that higher concentrations of bLf may induce similar level of apoptosis in MDA-MB-231 cells. A possible cause for this difference in behaviour may be due to the p53 status in the cell lines. MDA-MB-231 cells and MCF-7 cells differ in their p53 status with MDA-MB-231 cells harbouring a p53 mutation while MCF-7 are wild type. Differences in p53 modulation were observed in the apoptotic array with increases in phospho-p53 at serine 15 and 46 was observed with both Apo-bLf and Fe-bLf in MCF-7 cells while this was not observed in MDA-MB-231 cells. In fact, the opposite was observed in MDA-B-231 cells with all three p53 phosphorylation sites studies being downregulated following both Apo-bLf and Fe-bLf treatments. Given the complexity and importance of the p53 mechanism, extensive investigation into this finding could prove very interesting.

**Fig. 9:**
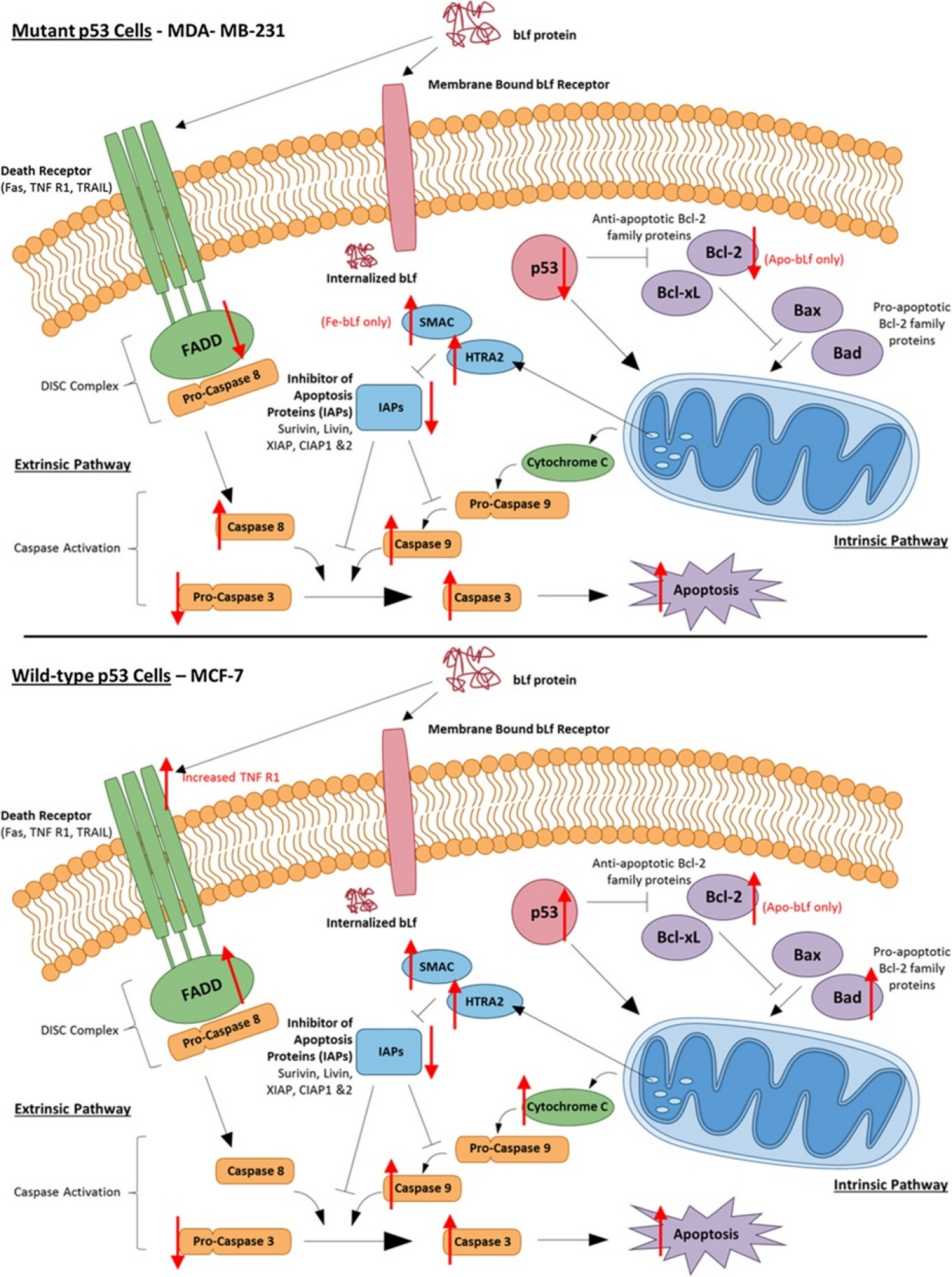
Apoptotic mechanisms in MDA-MB-231 and MCF-7 cells following treatment with Apo-bLf and Fe-bLf. BLf is internalized by membrane bound receptors such as Lf receptors via receptor mediated endocytosis. Once internalized, bLf modulates various apoptotic molecules as indicated by the red arrows. This modulation is cell specific as well as bLf form specific as some molecules are only activated or inhibited by one form of bLf

Survivin is a major inhibitor of apoptosis protein (IAP) acting in part by blocking the effect of caspases-3, −7 and −9 to allow cell evasion of apoptosis [1–3]. The role of native bLf, and its forms containing different levels of iron in targeting survivin expression in cancer cells is yet to be fully elucidated. More recently, we have reported that native bLf downregulates survivin gene expression in SW480 colon cancer cells [7], and Fe-bLf nanoformulation targets survivin to kill colon cancer stem cells [41] Here however it was found that MDA-MB-231 cells showed highly significant decreases in survivin protein expression levels, as well as strong increases in IAP inhibitors SMAC/DIABLO and HTRA2. SMAC/DIABLO was increased with Fe-bLf and HTRA2 was increased with both Apo-bLf and Fe-bLf. Furthermore, SMAC and HTRA2 were also upregulated in MCF-7 cells with both Apo-bLf and Fe-bLf. Both SMAC and HTRA2 inhibit IAPs by binding to them allowing caspase activation during apoptosis [49]. Western blot analysis of initiator caspases-8 and −9 as well as effector caspase-3 in MDA-MB-231 cells revealed large increases in cleavage of all three caspases, predominantly with Apo-bLf. This was in line with apoptosis levels determined via Annexin-V Fluos staining. The largest increase in caspase cleavage in MDA-MB-231 cells was with Apo-bLf in caspase-9 with an increase greater than 8-fold. This is consistent with the reduction in survivin given that survivin is known to bind and inhibit caspase-9 cleavage [50, 51]. Thus, apoptosis in MDA-MB-231 cells appears predominately through the modulation and suppression of IAPs, and increased activity of IAP inhibitors (Fig 9). It was also observed that cleaved caspases-8 and −9 were increased in MCF-7 cells following both Apo-bLf and Fe-bLf indicating that the increases observed in the IAP inhibitors could be contributing to the activation of these caspases.

Survivin expression was significantly reduced by bLf treatments at the protein level in both cell lines. Most notably, this is the first report of bLf reducing survivin in breast cancer cells and confirms our earlier findings in colon cancer cells. Both Apo-bLf and Fe-bLf have the capacity to reduce survivin protein expression demonstrated via Western blotting and protein array. Survivin downregulation was shown after 48 h in both cancer cell lines (Fig. 8) and after 24 h in MDA-MB-231 cells (Fig 5b). As compared to normal cells, survivin is overexpressed in almost all cancer types and considered a validated target for anti-cancer therapies [52], thus bLf shows great potential for this role. It is important to note that despite a number of survivin inhibitors being tested in clinical trials, none have passed this phase due to their toxicity issues where bLf has a proven safety profile [25, 30, 41].

Future investigations into the efficacy of Apo-bLf and Fe-bLf in *in vivo* models are of vital importance. Ideally, oral delivery is the preferred administration route due to the relative ease and patient compliance. Clinical trials in patients with colorectal polyps revealed a preventative potential of native bLf delivered daily (3.0 g) for 12 months [53, 54]. Furthermore, native bLf delivered in mouse and rat diet in cancer treatment models revealed reduction in tumour sizes in colon, esophageal, lung and [24, 55]. In addition, orally delivered Fe-bLf has been shown to augment the effects of traditional chemotherapeutics in breast and colon cancer models [24, 27]. These studies indicate that orally delivered bLf is a therapeutically viable delivery route. To further enhance the absorption and delivery of bLf, we have also reported the successful inhibition of xenograft tumours via oral delivery of nanoformulated bLf. Ceramic core, Fe-bLf encapsulated nanoparticles coated with chitosan and alginate were developed and delivered to mice bearing Caco-2 (colon cancer) xenograft tumours [25]. Tumour retardation was observed with successful delivery of Fe-bLf to the tumour site indicating that oral delivery of nanoformulated bLf may be a successful strategy to enhance absorption in the gut. Moreover, nanoformulated Fe-bLf was found to be highly significantly effective when given orally, as a pretreatment. This strategy could be applied to other cancers including breast cancer.

## Conclusions

In conclusion, it is reported that while both Apo-bLf and Fe-bLf showed anti-tumourigenic properties, Apo-bLf demonstrated an enhanced effect over Fe-bLf at inducing cytotoxicity and reducing invasion and migration in both cancer cell lines while Fe-bLf was more effective at inducing apoptosis in MCF-7 cell lines. This is a particularly novel finding given that iron saturated bLf has been found to be more efficient in colon cancer models in previous studies. Given that no significant cytotoxicity was detected in normal MCF-10-2A cells our findings highlight that bLf has great potential as a safe anti-cancer agent with cell-type specific effects. Furthermore, the modulation of different apoptotic molecules between MDA-MB-231 and MCF-7 cell highlights a multifunctional role of bLf in breast cancer cells. While it is evident that bLf ultimately induces caspase cleavage and finally apoptosis in both cancer cell lines, it is apparent that different pathways are being activated in the process, namely the IAP pathway in MDA-MB-231 cells and the p53 pathway in MCF-7 cells (Fig. 9).
