# Correction to: Iron-free and iron-saturated bovine lactoferrin inhibit survivin expression and differentially modulate apoptosis in breast cancer

**DOI:** 10.1186/s12885-018-4658-1

**Published:** 2018-07-19

**Authors:** Jessica A. Gibbons, Jagat R. Kanwar, Rupinder K. Kanwar

**Affiliations:** Nanomedicine - Laboratory for Immunology and Molecular Biomedical Research, Molecular and Medical Research Facility, School of Medicine, Faculty of Health, Deakin University, Geelong, Victoria Australia

## Correction to: BMC Cancer (2015) 15:425, DOI: 10.1186/s12885-015-1441-4

It has been highlighted that the original article [1] contains an error in Fig. 3 specifically, that it showed an incorrect version of Fig. 3c. This does not affect the Figure legend, results and conclusions of the article. The correct version of Fig. 3 is shown in this Correction article.

**Fig. 3:**
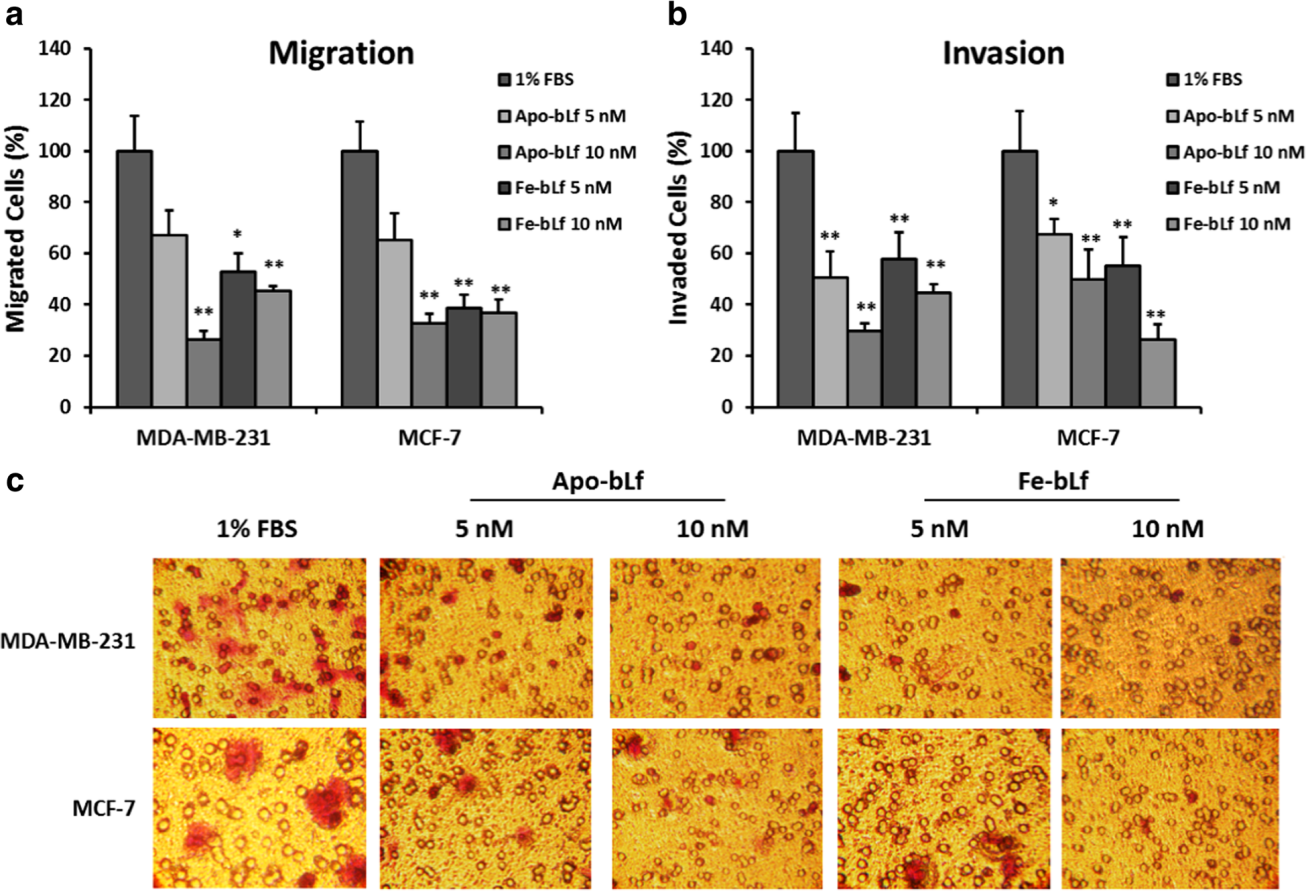
Effect on migration and invasion capacity of MDA-MB-231 and MCF-7 cells after treatment with bLf. Migration of MDA-MB-231 and MCF-7 (**a**) cells after bLf treatments for 24 h represented as a percentage of untreated (1% FBS) control migration. Invasion of MDA-MB-231 and MCF-7 cells (**b**) after bLf treatments for 24 h represented as a percentage of untreated (1% FBS) control invasion. * = *p* < 0.05 compared with 1% FBS group. Representative images (250X magnification) of invaded cells stained with crystal violet (**c**)
